# An Extracytoplasmic Function Sigma/Anti-Sigma Factor System Regulates Hypochlorous Acid Resistance and Impacts Expression of the Type IV Secretion System in *Brucella melitensis*

**DOI:** 10.1128/JB.00127-21

**Published:** 2021-05-20

**Authors:** Huoming Li, Sen Hu, Xin Yan, Yan Yang, Wenxing Liu, Zhigao Bu, Ganwu Li, Wentong Cai

**Affiliations:** a Key Laboratory of Veterinary Public Health of Ministry of Agriculture, State Key Laboratory of Veterinary Biotechnology, Harbin Veterinary Research Institute, Chinese Academy of Agricultural Sciences, Harbin, China; b Jiangsu Co-innovation Center for Prevention and Control of Important Animal Infectious Disease and Zoonoses, Yangzhou, China; Brigham and Women’s Hospital/Harvard Medical School

**Keywords:** extracytoplasmic function sigma factor, ECF, gene regulation, type IV secretion, virulence, *Brucella melitensis*, brucella, virulence regulation

## Abstract

The intracellular bacterial pathogen *Brucella* causes persistent infections in various mammalian species. To survive and replicate within macrophages, these bacteria must be able to withstand oxidative stresses and express the type IV secretion system (T4SS) to evade host immune responses. The extracytoplasmic function (ECF) sigma factor system is a major signal transduction mechanism in bacteria that senses environmental cues and responds by regulating gene expression. In this study, we defined an ECF σ *bcrS* and its cognate anti-σ factor *abcS* in Brucella melitensis M28 by conserved domain analysis and a protein interaction assay. BcrS directly activates an adjacent operon, *bcrXQP*, that encodes a methionine-rich peptide and a putative methionine sulfoxide reductase system, whereas AbcS is a negative regulator of *bcrS* and *bcrXQP*. The *bcrS*-*abcS* and *bcrXQP* operons can be induced by hypochlorous acid and contribute to hypochlorous acid resistance *in vitro*. Next, RNA sequencing analysis and genome-wide recognition sequence search identified the regulons of BcrS and AbcS. Interestingly, we found that BcrS positively influences T4SS expression in an AbcS-dependent manner and that AbcS also affects T4SS expression independently of BcrS. Last, we demonstrate that *abcS* is required for the maintenance of persistent infection, while *bcrS* is dispensable in a mouse infection model. Collectively, we conclude that BcrS and AbcS influence expression of multiple genes responsible for *Brucella* virulence traits.

**IMPORTANCE** *Brucella* is a notorious intracellular pathogen that induces chronic infections in animals and humans. To survive and replicate within macrophages, these bacteria require a capacity to withstand oxidative stresses and to express the type IV secretion system (T4SS) to combat host immune responses. In this study, we characterized an extracytoplasmic function sigma/anti-sigma factor system that regulates resistance to reactive chlorine species and T4SS expression, thereby establishing a potential link between two crucial virulence traits of *Brucella*. Furthermore, the anti-sigma factor AbcS contributes to *Brucella* persistent infection of mice. Thus, this work provides novel insights into *Brucella* virulence regulation as well as a potential drug target for fighting *Brucella* infections.

## INTRODUCTION

Brucellosis, also known as Malta fever, is one of the most notorious zoonotic diseases, and it impacts many mammalian species worldwide. The disease is induced by the Gram-negative facultative intracellular bacteria *Brucella*, a genus that contains at least 10 recognized species (1, 2). Brucellosis in animals usually leads to abortions and infertility, while brucellosis in humans is characterized by extreme fatigue and undulant fever (3). In either case, the bacteria can survive and are maintained for a long time in various organs, such as the spleen, liver, and bone marrow. The long-term persistence of *Brucella* relies on its capacity to withstand oxidative stress encountered within host macrophages and neutrophils (4–6). Multiple systems involved in oxidative stress resistance and virulence *in vivo* have been described, including the superoxide dismutase SodC (7), the regulators Irr (8) and MucR (9), and the twin-arginine translocation system (10).

Despite the lack of many classical virulence factors, such as toxins and fimbriae, *Brucella* possesses the *virB* locus that encodes a type IV secretion system (T4SS) (11). T4SSs are evolutionarily related to bacterial conjugation systems and have been shown to contribute to virulence in a variety of bacterial pathogens (12–14). In *Brucella*, the VirB T4SS locus contains 12 genes, namely, *virB1* to *virB12*, transcribed from a single promoter located before *virB1*. T4SS is required for intracellular survival and replication of *Brucella* in different cell models (15, 16), and it is essential for *Brucella* chronic infection in murine and caprine models (17–19). *Brucella* T4SS is regulated in response to relevant host-derived stimuli. For instance, low-pH conditions within *Brucella*-containing vacuoles arising from acidification when fusing with lysosomes can induce the expression of the *virB* operon (11, 72, 73). Other T4SS regulators include the two-component signaling system (TCS) BvrR/BvrS (20), quorum-sensing regulator VjbR (21), and histidine utilization regulator HutC (22). More T4SS regulators likely remain to be identified, and links between T4SS regulators and relevant *in vivo* conditions are largely unknown.

To initiate transcription at promoters, bacterial RNA polymerase (RNAP) requires a sigma factor (σ), which is responsible for the direct recognition of promoter elements and stimulation of initial steps in RNA synthesis (23). Sigma factors can be categorized into two major families according to their similarity to two σ factors in Escherichia coli: the primary σ^70^ family, which directs mainly the transcription of housekeeping genes, and the structurally unrelated σ^54^ family, which is responsible for gene transcription in response to environmental cues (24). σ^70^ family members contain up to four structurally conserved domains (σ_1_ to σ_4_), and based on the presence or absence of these conserved domains and regions, they are classified into four distinct groups, i.e., groups 1 to 4. Group 4 alternative σ factors, also known as extracytoplasmic function (ECF) σ factors, lack both σ_1_ and σ_3_ and contain only σ_2_ and σ_4_ domains. During the process of DNA double-strand separation, σ_2_ performs base-specific interactions with the −10 promoter element, while σ_4_ employs a helix-turn-helix motif to bind to the −35 element (25, 26).

ECF σ systems typically sense and respond to changes in extracytoplasmic compartments of the cell, such as oxidative and osmotic stresses, temperature shifts, and nutrient concentrations (27). Therefore, ECF σ system represents one of the two major mechanisms used by bacteria to transduce extracellular signals to the cytoplasm, with the other being TCS. ECF σ factors frequently form an operon with their cognate regulators (anti-σ) and autoregulate their own expression (28). Anti-σ factors are usually inner membrane-bound and bind to σ factors to prevent their association with the RNAP core enzyme (29). Extracytoplasmic signals trigger a proteolytic cascade leading to degradation of the anti-σ factor and subsequent release of the σ. The released σ factor binds to the RNAP core and initiates transcription (30). ECF σ factors have been implicated in the pathogenesis of many bacterial pathogens, such as σ^E^ in *Brucella* (31–34).

The ECF16 subgroup, which is particularly present in alphaproteobacteria, is related to SigF from Caulobacter crescentus, which mediates the oxidative stress response. A unique characteristic of ECF16 σ is its anti-σ factor, which contains a DUF1109 domain with six transmembrane regions (35). Here, we found that in addition to the conserved ECF σ^E^ (31), *Brucella* spp. encode an ECF16 σ/anti-σ system, and this system is encoded by B0022/B0023 in the strain B. melitensis M28. Furthermore, the target genes, biological functions, molecular regulatory mechanisms, and pathogenic roles of this system were determined. Based on their contributions to resistance to reactive chlorine species (RCS), B0022 was designated *bcrS* (*Brucella* RCS resistance sigma factor), B0023 was designated *abcS* (anti-*Brucella* RCS resistance sigma factor), and B0021-B0019 was designated *bcrXQP*.

## RESULTS

### Identification of an ECF σ and anti-σ system in *B. melitensis*.

**FIG 1:**
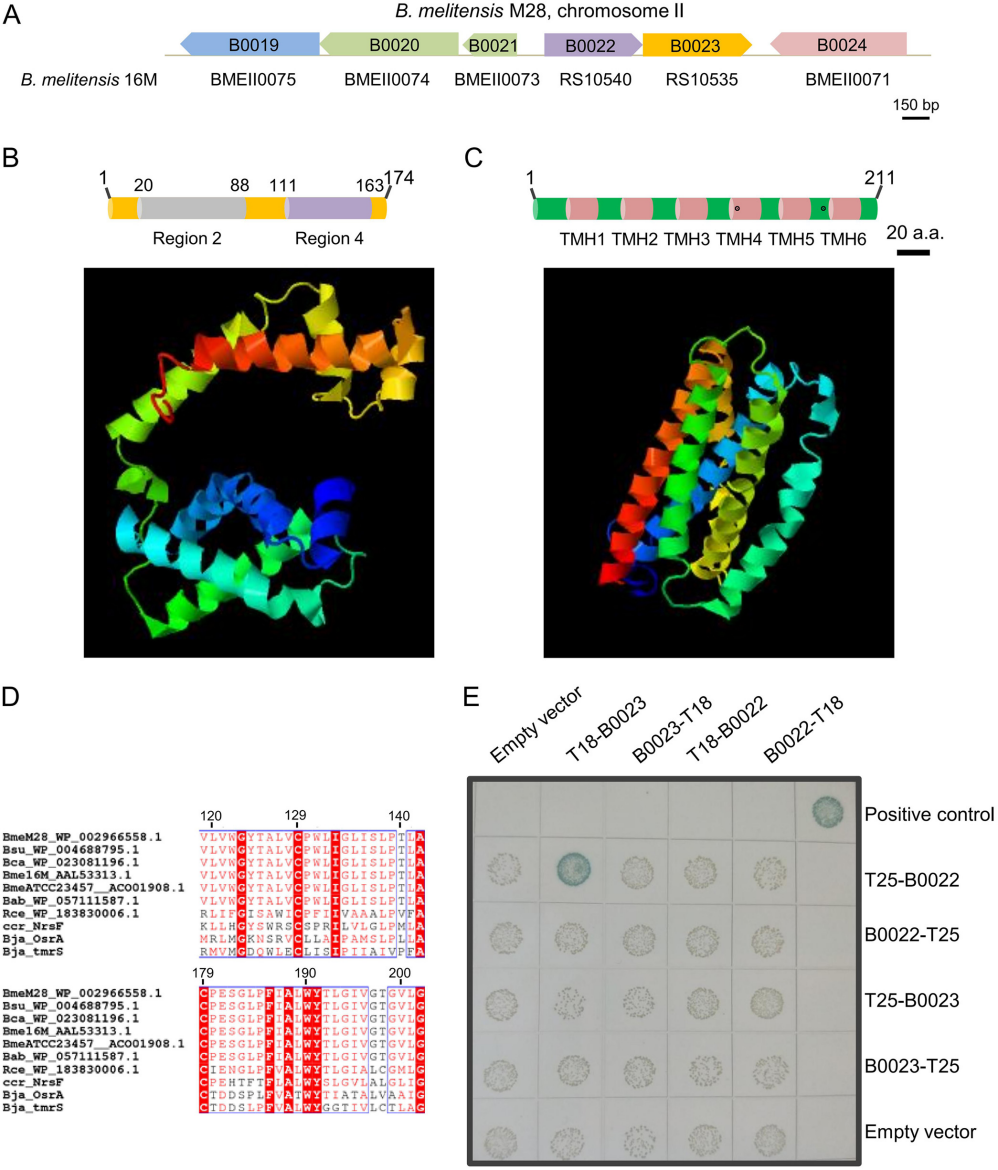
Identification of an ECF σ (B0022) and its cognate anti-σ factor (B0023) in B. melitensis M28. (A) Genetic organization of B0022 and B0023. B0021 encodes a methionine-rich MXKDX repeat protein, and B0024 encodes a GntR family transcriptional regulator. Locus tags of the corresponding genes in B. melitensis 16M (accession number NC_003318) are shown under the arrows. The 3-D structures of B0022 (B) and B0023 (C) were predicted by the I-TASSER online server with default parameters, and the domain architectures are illustrated, along with their structures. Domains σ_2_ and σ_4_ usually carried by ECF σ factors are shown. The transmembrane helices (TMH1 to TMH6) in B0023 were predicted by the transmembrane protein topology prediction tool TMHMM. Two conserved cysteine residues (C129 and C179) suggested to be involved in binding to σ and signal response are depicted as black dots. Numbers on the domain architectures indicate the positions of amino acid residues. (D) Partial sequence alignment of anti-σ B0023 and its homologs. The alignment was created using ClustalW. The two conserved cysteine residues suggested to be involved in binding to σ and signal response are located at positions 129 and 179. Red letters, similar amino acids; shaded letters, conserved amino acids; black letters, not conserved. Source strains are as follows: Brucella suis (Bsu), Brucella canis (Bca), Brucella melitensis 16M (Bme16M), Brucella melitensis ATCC 234567 (BmeATCC23457), Brucella abortus (Bab), Rhizobium cellulosilyticum (Rce), Caulobacter crescentus (Ccr), Bradyrhizobium japonicum (Bja), and Brucella melitensis *M28* (BmeM28). (E) Assessment of the B0022-B0023 interaction using a bacterial two-hybrid system. Adenylate cyclase fragments T18 and T25 of Bordetella pertussis were translationally fused to B0022 and B0023 at their N and C termini, respectively. Two plasmids in each combination were cotransformed into the E. coli BTH101 strain, and properly diluted transformants were spotted on agar plates with X-Gal. As a positive control, plasmids encoding T25-zip and T18C-zip with known interactions were used. Blue colonies indicate substantial LacZ activities, suggesting that protein interaction takes place; white colonies indicate no LacZ activities, suggesting no detectable protein interaction. Experiments were repeated three times, and a representative image is shown.

*Brucella* spp. are highly adaptive to mammalian hosts (36). The ECF system is a major mechanism used by bacteria to elicit an adaptive response to extracytoplasmic signals (26). Our bioinformatic analysis of σ factors in B. melitensis M28 identified the σ^70^ family σ factor B0022, comprising 174 amino acids (aa), and its downstream DUF1109 family protein B0023, with 211 aa (Fig. 1A). The B0022/B0023 locus can also be found in other species of *Brucella*, including B. abortus and B. suis. Further domain analysis showed that B0022 contains two conserved domains of σ^70^ family members, namely, region 2 (aa 20 to 88, IPR013325), which is usually engaged in binding to the −10 motif and core RNAP, and region 4 (aa 111 to 163, IPR013324), which is usually used for binding to the −35 motif. In addition, a predicted three-dimensional (3-D) structure displayed two distinct domains (Fig. 1B). Thus, B0022 belongs to group 4 of the σ^70^ family, i.e., the ECF group. B0022 is 45% identical to ECF SigF, which is involved in resistance to oxidative stress in C. crescentus.

Domain and TMHMM transmembrane region analysis indicated that B0023 contains six transmembrane helices, and this architecture was also reflected in the predicted 3-D structure (Fig. 1C). B0023, belonging to the anti-σ NrsF family (IPR009495), is 31% identical to the anti-σ OsrA responsible for resistance to reactive oxygen species in Bradyrhizobium japonicum, suggesting that B0023 is an ECF16 anti-σ. A sequence alignment of ECF16 anti-σ factors revealed that B0023 contains two conserved cysteine residues (Fig. 1D), C129 and C179. C129 is critical for maintaining anti-σ in an interaction-competent conformation; and periplasm-exposed C179 likely detects a signal directly and transduces it to the cytoplasmic segment of the anti-σ, where it leads to the release of the bound σ (37, 38).

If B0022 and B0023 function as a typical cognate σ/anti-σ duo, they would bind to each other at the protein level. We thus used an adenylate cyclase-based bacterial two-hybrid system to examine the direct interaction between B0022 and B0023. B0022 and B0023 were each fused to adenylate cyclase subdomains T18 and T25 at their N and C termini. The LacZ activity results showed that only the combination of T18-B0023 and T25-B0022 produced blue colonies, which is indicative of induction of β-galactosidase activities, while the negative controls produced the expected white colonies (Fig. 1E). These data indicate that the direct interaction of B0022 and B0023 allowed the functional complementation of the T18 and T25 subdomains. Altogether, our data establish that B0022 and B0023 encode ECF16 cognate σ/anti-σ.

### B0022 (BcrS) and B0021-B0019 (BcrXQP) contribute to HOCl resistance.

**FIG 2:**
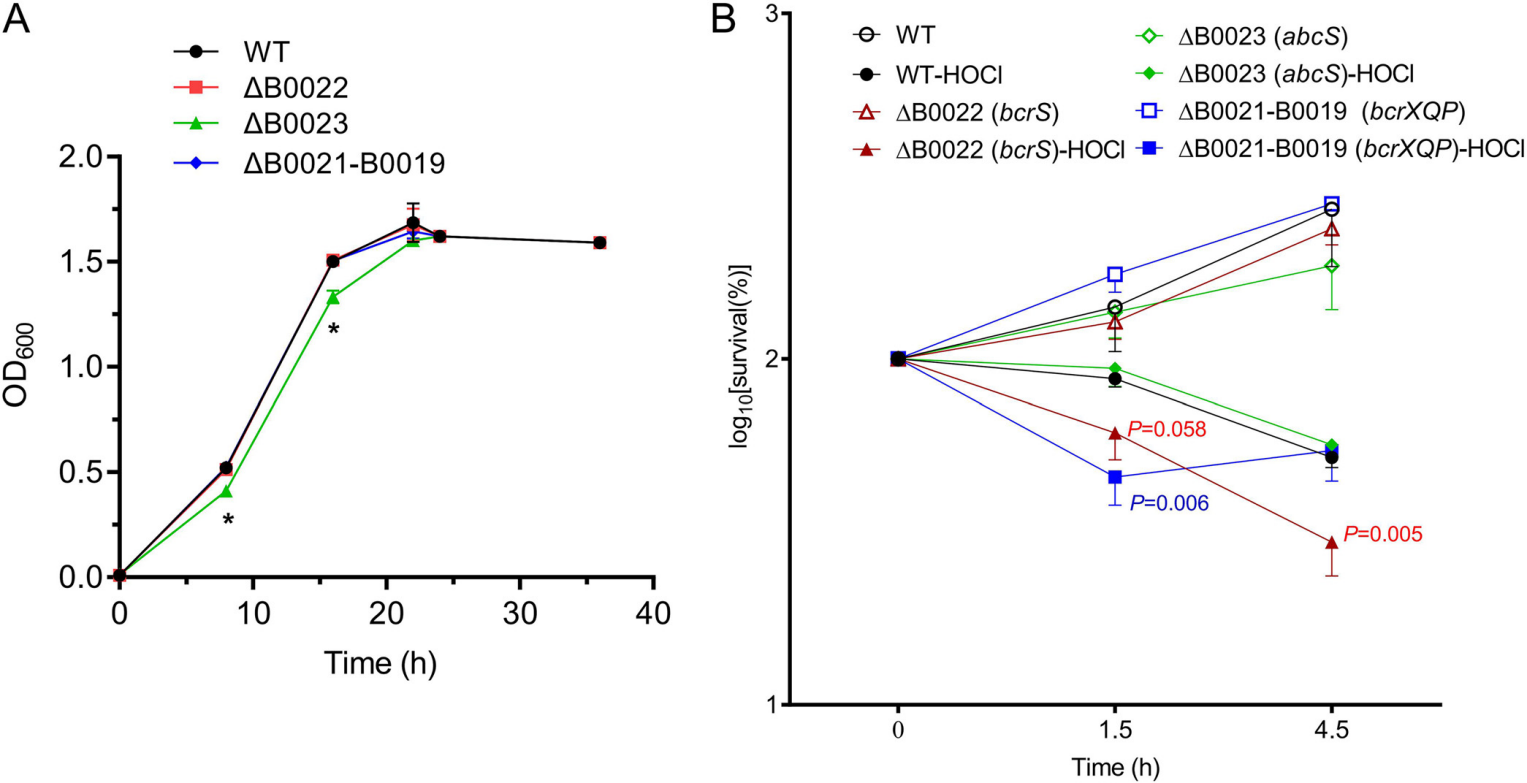
Roles of B0022, B0023, and B0021-B0019 in the resistance to HOCl. (A) Growth curves of M28 and its mutant strains. Bacteria were grown in TSB medium, and the optical density was measured at the indicated time points. Data were collected from three replicates. *, *P* < 0.05 (one-way ANOVA, followed by Dunnett’s multiple-comparison test). (B) Wild-type M28 and its mutant strains were grown in TSB medium until reaching an OD_600_ of ∼0.5, and then bacterial cultures were aliquoted into two portions, with one treated with HOCl at a final concentration of 2 mM and the other with PBS as an untreated control. Samples were taken at the indicated times, diluted with PBS, and plated on TSA plates for CFU counts. The CFU counts at time point 0 were set as 100%. The percentage of survival was calculated as (CFU at a time point after stress/CFU at time point 0) × 100%. Experiments were repeated three times, and values are the means ± the standard errors of the mean (SEM). One-way ANOVA, followed by Dunnett’s multiple-comparison test, was used to evaluate significant differences between the mutants and the wild type, and a *P* value of <0.05 was considered statistically significant. Gene names in parentheses are used here.

B0021, encoding a methionine-rich MXKDX repeat protein, is immediately upstream of B0022 and homologous to MsrX in *Azospira suillum*, which scavenges hypochlorites by sacrificial oxidation of itself (39). B0020-B0019 immediately upstream of B0021 encodes proteins homologous to the E. coli methionine sulfoxide (MetSO) reductase system MsrQP, which reduces MetSO to methionine in proteins under HOCl stress (40). To test the roles of B0022-B0023 and B0021-B0019 in *Brucella* resistance to HOCl and H_2_O_2_, single mutants ΔB0022 and ΔB0023 and triple mutant ΔB0021-B0019 were constructed and subjected to HOCl and H_2_O_2_ survival assays. We first determined the growth curves of the wild type (WT) and its mutants in tryptic soy broth (TSB) medium. The results demonstrated that the ΔB0022 and ΔB0021-B0019 mutants displayed similar growth kinetics compared to the wild type; and ΔB0023 mutant grew slightly slower than the wild type during the exponential phase (*P* < 0.05), and yet it caught up with the wild type as these entered the stationary phase (Fig. 2A). Figure 2B shows that the survival of ΔB0021-B0019 at 1.5 h was significantly lower in the presence of 2 mM HOCl compared to that of the wild type (*P* < 0.01), whereas the survival of ΔB0022 and ΔB0023 was statistically indistinguishable from that of the wild type. At 4.5 h after stress, the survival of the ΔB0022 mutant was lower than that of the other strains (*P* < 0.01). On the other hand, the survival of all strains was comparable upon treatment with H_2_O_2_ (data not shown). Therefore, these results show that B0022 and B0021-B0019 contribute to the resistance of B. melitensis to HOCl. Thus, we renamed B0022 *bcrS* (*Brucella* RCS resistance sigma factor), B0023 *abcS* (anti-*Brucella* RCS resistance sigma factor), and B0021-B0019 *bcrXQP*.

### The *bcrXQP* operon can be induced by HOCl and is directly regulated by *bcrS*-*abcS*.

**FIG 3:**
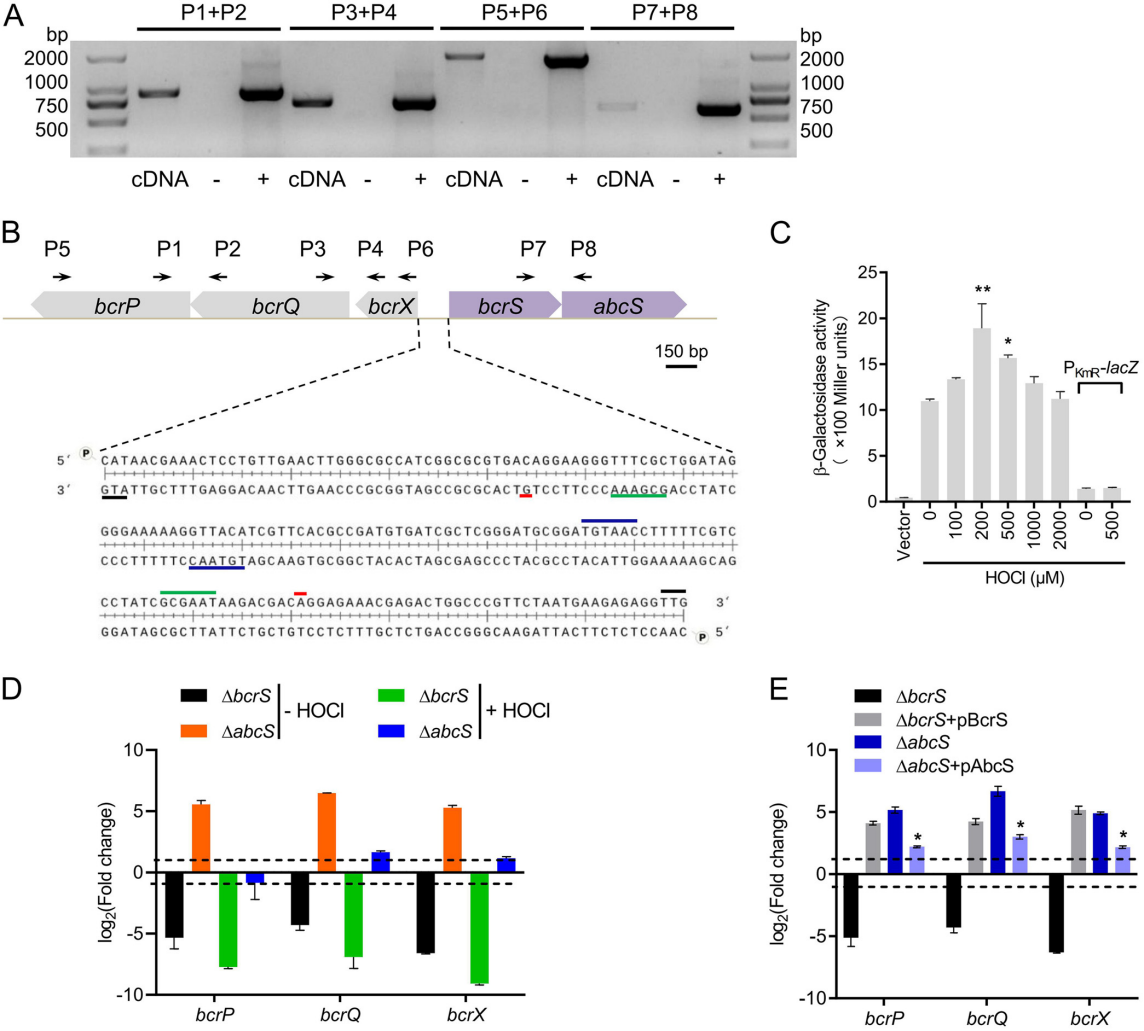
*bcrXQP* operon can be induced by HOCl and is regulated by *bcrS*-*abcS*. (A) Operon formation of *bcrXQP* and *bcrS*-*abcS* examined by RT-PCR. RNA was isolated from B. melitensis M28 grown in TSB and reverse transcribed to cDNA. RNA that was not reverse transcribed served as a negative control, while genomic DNA served as a positive control. Primers P1 to P8, whose positions are displayed in Fig. 3B, were designed to span *bcrP* and *bcrQ*, *bcrQ* and *bcrX*, *bcrP* to *bcrX*, and *bcrS* and *abcS*. For each primer pair, the three lanes represent cDNA, negative control, and positive control. (B) Genetic organization and characteristics of the *bcrXQP* and *bcrS*-*abcS* promoters. The 5′-RACE method was applied to identify the TSSs of the *bcrXQP* and *bcrS*-*abcS* operons using RNA samples from Fig. 3A, and the −10 and −35 motifs were deduced afterward. Black, red, green, and blue lines amid the DNA sequences represent the start codon, TSS, −10, and −35 motifs, respectively. Genes were drawn to scale. (C) Expression of *P_bcrX_*-*lacZ* in response to different concentrations of HOCl. Bacteria were cultured in TSB broth until reaching an OD_600_ of ∼0.5, and the culture was then aliquoted into multiple portions for 60 min of stimulation with different concentrations of HOCl. An empty vector carrying promoterless *lacZ* was used as a vector control, and P_KmR_-*lacZ* was used as an uninducible control. Values are the means ± the SEM of triplicate samples from three independent experiments. Significance was analyzed by one-way ANOVA, followed by Dunnett’s multiple-comparison test. *, *P* < 0.05; **, *P* < 0.01. (D and E) Regulatory roles of *bcrS* and *abcS* in the expression of *bcrXQP*. RNA samples from the wild type, various mutants and complemented strains were prepared as in Fig. 3C, and HOCl was present at 1 mM for 10 min when needed. qPCR was carried out to compare gene expression levels. The gene encoding 16S rRNA was used as an internal control, and the 2^–ΔΔ^*^CT^* method was used to determine fold changes in the mutants and complemented strains compared to the wild type. Dashed lines represent a 2-fold change cutoff, and a fold change of ≥2 was considered significant. *, significant difference in gene expression between the Δ*abcS*+pAbcS strain and the Δ*abcS* strain.

Homologs of *bcrXQP* in the soil bacterium *A*. *suillum*, namely, *yedYZ*-*mrpX*, are functionally and transcriptionally linked (39). We went on to test whether *bcrXQP* is transcribed as one transcription unit using reverse transcription-PCR. Primers were designed to span two adjacent genes, as well as all three genes. As shown in Fig. 3A, specific amplicons were produced that spanned *bcrP*-*bcrQ*, *bcrQ*-*bcrX*, and *bcrP*-*bcrQ*-*bcrX* using cDNA as the templates. No amplicons were obtained in the negative-control reactions, and a strong band was observed in the positive-control reaction mixture containing M28 genomic DNA (Fig. 3A). These results suggest that *bcrX*, *bcrQ*, and *bcrP* are cotranscribed. To identify the transcription start site (TSS) of *bcrXQP*, 5′ rapid amplification of cDNA ends (5′-RACE) was performed (see Fig. S1 in the supplemental material). The TSS and the deduced −10 and −35 motifs for the *bcrXQP* operon are shown in Fig. 3B. The −10 (GCGAAA) and −35 (TGTAAC) motifs are highly concordant with the ECF16 σ consensus recognition sequence [(T/C)GTAAC-N_17_-CGAA] (35).

To determine whether the transcription of P*_bcrXQP_* responded to the presence of HOCl, a P*_bcrXQP_*-*lacZ* transcriptional fusion was created on the pBBR plasmid, and the resultant construct was transformed into wild-type M28. Figure 3C shows that the presence of 200 μM and 500 μM HOCl significantly upregulated the expression of P*_bcrXQP_*-*lacZ*, although a further increase in HOCl concentration did not lead to stronger upregulation of P*_bcrXQP_*-*lacZ* expression, possibly due to the strong oxidative effects of HOCl. A control plasmid carrying constitutively expressed P_KmR_-*lacZ* fusion was not responsive to the addition of HOCl. These results demonstrate that the *bcrXQP* operon can be induced by HOCl.

Next, we sought to characterize the roles of *bcrS* and *abcS* in *bcrXQP* transcription. The mRNA levels of *bcrXQP* in the wild type, in Δ*bcrS* and Δ*abcS* mutants, and in corresponding complemented strains in the absence or presence of HOCl were investigated using qPCR. Deletion of *bcrS* caused a drastic reduction (>16-fold) in *bcrXQP* transcription levels relative to the wild type, whereas in the presence of HOCl, the reduction was even greater (at least 2-fold reduction from that in the absence of HOCl). Lacking *abcS* dramatically augmented the *bcrXQP* transcription levels (Fig. 3D). A plasmid-borne *bcrS* locus increased *bcrXQP* transcription levels in the Δ*bcrS* mutant, and the levels were even higher than that in the wild type; in addition, an *abcS* locus encoded on pBBR plasmid significantly decreased *bcrXQP* transcription in the Δ*abcS* mutant, albeit not to the wild-type levels (Fig. 3E). Collectively, these data suggest that BcrS directly activates the expression of *bcrXQP* and that AbcS is a negative regulator of *bcrXQP*.

### The *bcrS* operon is inducible by HOCl and autoregulated.

**FIG 4:**
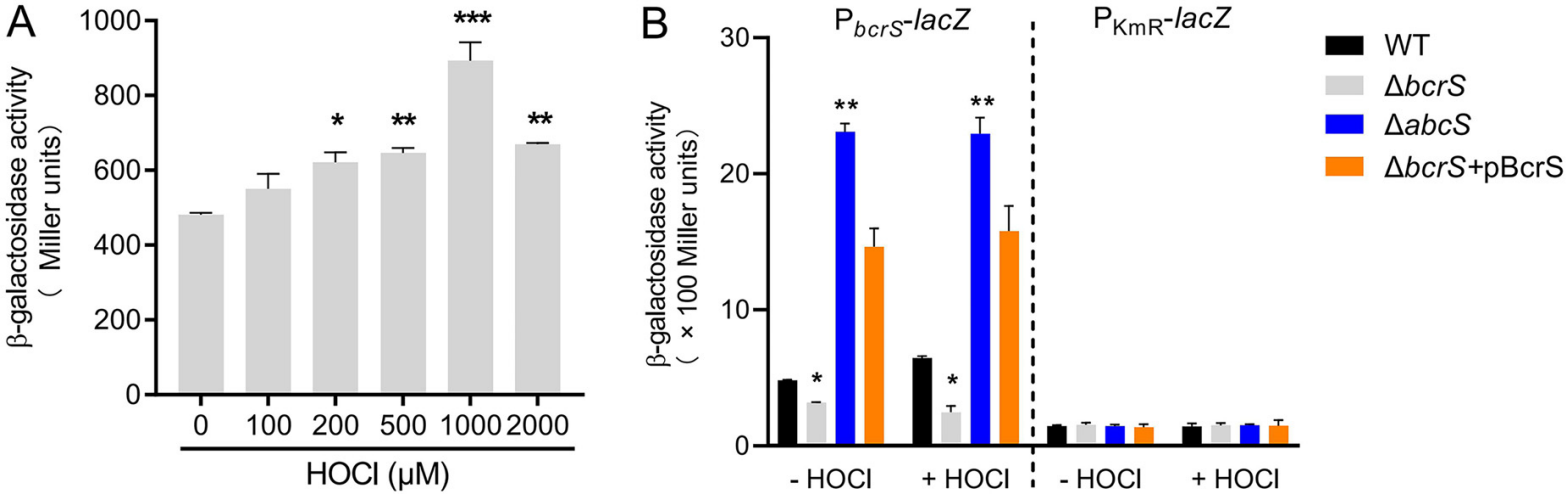
*bcrS* operon can be induced by HOCl and is autoregulated. (A) M28 carrying a pBBR plasmid with P*_bcrS_*-driven *lacZ* was stimulated by HOCl (1 mM) for 60 min, and then bacterial cells were lysed for β-galactosidase activity measurement. (B) Expression of P*_bcrS_*-*lacZ* in the wild-type M28, Δ*bcrS* mutant, Δ*bcrS* complemented strain, and Δ*abcS* mutant in the absence or presence of HOCl (1 mM). Sample preparation and β-galactosidase activity assays were performed as shown in Fig. 3C. Significance was analyzed by one-way ANOVA, followed by Dunnett’s multiple-comparison test. *, *P* < 0.05; **, *P* < 0.01; ***, *P* < 0.001.

RT-PCR demonstrated that *bcrS* and *abcS* are cotranscribed (Fig. 3B), and 5′-RACE identified the TSS of the *bcrS*-*abcS* operon (see Fig. S1 in the supplemental material). The −10 (GCGAAT) and −35 (TGTAAC) motifs were deduced accordingly, and they are highly similar to those in the P*_bcrXQP_* promoter region (Fig. 3B). To examine whether *bcrS* expression was induced in response to HOCl, a plasmid-borne P*_bcrS_*-*lacZ* fusion construct was generated and introduced into M28, and the β-galactosidase activities were assayed in the presence of various concentrations of HOCl. Figure 4A shows that as the HOCl concentrations increased, the expression of P*_bcrS_*-*lacZ* clearly exhibited an increasing trend and peaked when HOCl was present at 1 mM. These results indicate that the *bcrS*-*abcS* operon is inducible by HOCl.

A number of ECF σ factors autoregulate their own expression, which likely occurs to augment their responses to extracytoplasmic stimuli (28, 35). Since we identified BcrS recognition sequences upstream of the *bcrS* coding region, we hypothesized that *bcrS* was autoregulated. To test this, P*_bcrS_*-*lacZ* expression was determined in the wild type, Δ*bcrS* and Δ*abcS* mutants in the absence and presence of HOCl. Notably, the qPCR analysis suggested that replacement of *bcrS* by the KmR marker did not greatly affect the expression of *abcS* compared to the wild type, which was probably due to the readthrough effects of the KmR cassette (data not shown). As shown in Fig. 4B, in the absence of the inducer HOCl, P*_bcrS_*-*lacZ* expression was significantly downregulated in the Δ*bcrS* mutant, whereas P*_bcrS_*-*lacZ* expression was upregulated by ∼5-fold in the Δ*abcS* mutant compared to the wild type. As expected, introduction of the *bcrS* locus into the Δ*bcrS* mutant substantially increased P*_bcrS_*-*lacZ* expression. Similar trends were obtained when HOCl was present. Moreover, the expression of P_KmR_-*lacZ* was not responsive to the addition of HOCl or deletion of *bcrS* or *abcS*. Together, these data demonstrate that the *bcrS* operon can be induced by HOCl and is autoregulated and further corroborate that *abcS* is a negative regulator of *bcrS*. These results were consistent with our transcriptome sequencing (RNA-seq) analysis (see below).

### Identification of genes regulated by BcrS/AbcS using RNA-seq.

**FIG 5:**
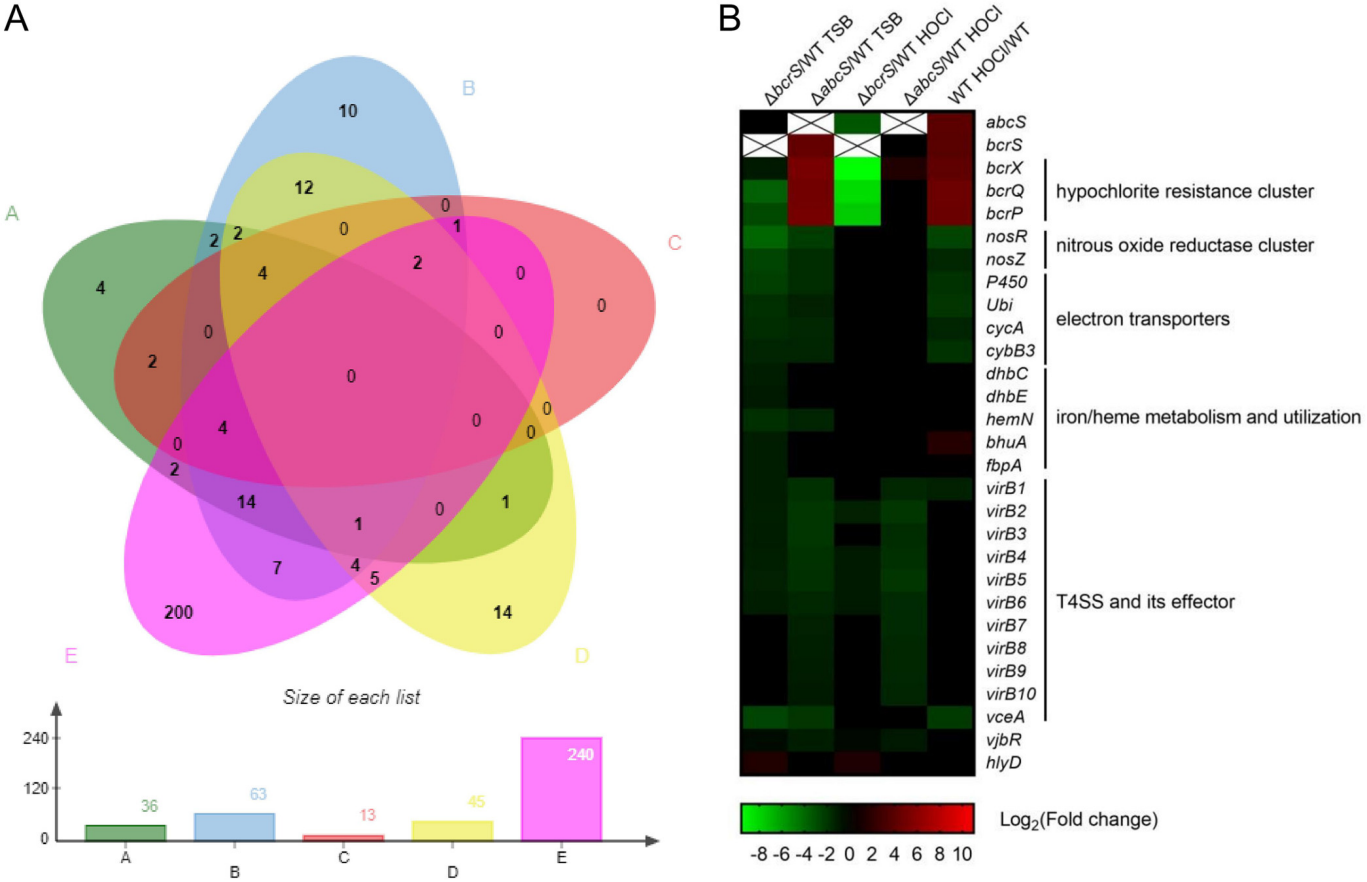
Identification of genes regulated by BcrS and AbcS using RNA-seq. (A) Venn diagram representing shared and unique differentially expressed genes among the five groups. The bar graph below shows the numbers of differentially expressed genes in each group. Groups A to E represent Δ*bcrS* versus WT in TSB, Δ*abcS* versus WT in TSB, Δ*bcrS* versus WT in HOCl, Δ*abcS* versus WT in HOCl, and WT induced by HOCl versus WT noninduced, respectively. (B) Heatmap demonstrating a selection of genes differentially expressed in the comparisons indicated above each column. Gene names and functional categories are shown on the right. The graph was created based on the mean value of fold changes in triplicates. The “cross” means those data are not applicable.

To gain a much deeper understanding of the *bcrS*-*abcS* system, we wanted to identify all the genes whose transcription might be regulated by *bcrS* and *abcS*. Total RNA from the wild type (WT) and Δ*bcrS* and Δ*abcS* mutants grown with or without HOCl was isolated, and their transcriptomes were obtained by RNA sequencing. A 2-fold change was selected as the cutoff. Comparative transcriptomic analysis indicated that 36, 63, 13, 45, and 240 genes were differentially expressed in the Δ*bcrS* versus WT in TSB (group A), Δ*abcS* versus WT in TSB (group B), Δ*bcrS* versus WT in HOCl (group C), Δ*abcS* versus WT in HOCl (group D), and WT HOCl versus WT (group E) binary comparisons, respectively (Fig. 5A; Tables S1 to S5 list differentially expressed genes [DEGs] in the five groups). Interestingly, deletion of *abcS* affected more genes than deletion of *bcrS* (group B versus group A and group D versus group C) and 75% of DEGs in group A were also upregulated in group B. More genes were affected by *bcrS* deletion in the absence of the inducer HOCl than in the presence of it (group A versus group C, Fig. 5A). The *bcrXQP* and *bcrS*-*abcS* operons were markedly induced by HOCl and were among the most upregulated genes in response to HOCl. With or without HOCl, *bcrS* positively regulated the transcription of *bcrXQP*, whereas *abcS* negatively regulated the transcription of *bcrXQP* (Fig. 5B). These data therefore support our previous conclusions (Fig. 3C and D; Fig. 4A and B).

Figure 5B displays a great majority of genes from groups A and C, excluding several that are functionally unknown or irrelevant to virulence (see Tables S1 to S5 in the supplemental material). Nearly all of these genes are located on chromosome II (∼95%). Specifically, RS11395 (the BMEII1002 ortholog predicted to encode a hemerythrin domain-containing protein that binds to calcium/iron), *hemN* (encoding a heme maturation protein), *bhuA* (encoding a heme transporter), RS13430 (the BMEII0584 ortholog encoding the periplasmic component of an *fbpA* ABC-type Fe^3+^ transport system), and *dhbCE* (encoding 2,3-dihydroxybenzoic acid [2,3-DHBA] siderophore biosynthesis enzymes) are related to iron/heme/siderophore metabolism. *nosR* and its downstream *nosZ* are part of a cluster responsible for nitrous oxide reductase production. The *ubiDX* operon (involved in ubiquinone biosynthesis), *cycA* (encoding a cytochrome *c* family protein), *cybB3* (encoding a cytochrome *b* family protein), *azu1* (encoding pseudoazurin), and RS11975 (encoding a cytochrome P450-containing protein) are related to electron transporters. Compared to the wild type, genes encoding T4SS VirB1 to VirB6 components and *vceA* encoding a T4SS effector were uniformly downregulated by at least 2-fold (log_2_fold change < −1) in the Δ*bcrS* mutant, while 10 T4SS genes (*virB1* to *virB10*) plus *vceA* were uniformly downregulated by 2- to 4-fold in the Δ*abcS* mutant.

### Genome-wide search of BcrS recognition sequences.

**FIG 6:**
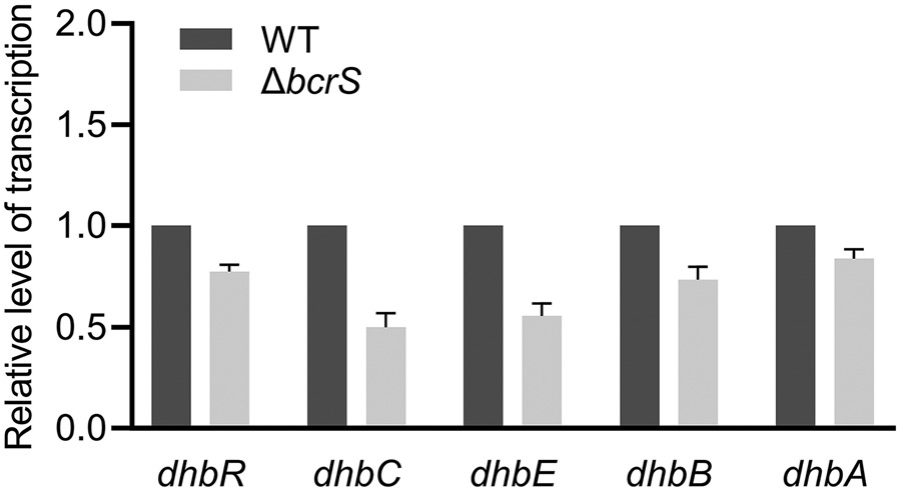
Contribution of *bcrS* to the expression of genes related to iron utilization. Bacteria were cultured in TSB broth without HOCl until reaching an OD_600_ of ∼0.5, and then RNA samples were prepared. The relative transcription levels were calculated compared to the 16S rRNA gene as an internal control, and the levels in the wild type were set as 1.0. Values are the means ± the SEM from three independent experiments.

To identify more BcrS recognition sequences and potentially discover additional direct targets of BcrS, we carried out a genome-wide search of BcrS recognition sequences composed of the −10 and −35 motifs. To this end, the GTAA-N_14–21_-CGAA consensus was searched in the M28 genome using the Pattern Locator program (41). Table 1 lists the output of 14 entries, including the recognition sequences of *bcrX* and *bcrS*. Two sequences upstream of genes encoding a lytic murein transglycosylase and a MerR-family transcriptional regulator are exceedingly similar to those of *bcrX* and *bcrS*. Nonetheless, transcription of these two genes did not respond to deletion of *bcrS* in our RNA-seq analysis. *dhbR*, which has a recognition sequence in its promoter region, encodes a regulator of *dhbCEBA* responsible for 2,3-DHBA siderophore biosynthesis (42). *dhbCE* were downregulated in the *bcrS* mutant (Fig. 5B). We further used qPCR to determine the *dhbR* expression levels, and the results showed that *dhbR* expression was slightly decreased in the *bcrS* mutant, with a <2-fold change (Fig. 6). Thus, BcrS likely directly impacts *dhbR* expression, which in turn alters the expression of its target genes, *dhbCEBA*. Altogether, our data identified at least three BcrS direct targets: *bcrX*, *bcrS*, and *dhbR*.

**TABLE 1:** List of potential BcrS recognition sequences identified by the Pattern Locator program

Location	Start	End	Strand	Recognition sequence (−35 and −10 motifs)^*a*^	Downstream	Functional annotation
Chromosome II	18520	18545	–	CGAT**GTAA**CCTTTTTCCCCTATCCAG**CGAA**ACCCTT	BM28_RS10385	Methionine-rich peptide BcrX
Chromosome II	18584	18609	+	GGAT**GTAA**CCTTTTTCGTCCCTATCG**CGAA**TAAGAC	BM28_RS10390	ECF BcrS
Chromosome I	339024	339051	+	GCCG**GTAA**CGAACCGCGGCCTTATTTAG**CGAA**TTCTTT	BM28_RS17565	Hypothetical protein
Chromosome I	1180795	1180821	–	TTGT**GTAA**GATATAAATTTGCAAATAG**CGAA**ATGCGA	BM28_RS05685	Transcriptional regulator MerR
Chromosome I	1249151	1249173	–	CTCTC**GTAA**TAGGTTGGTGAAGCA**CGAA**ACTTGT	BM28_RS06055	Conserved periplasmic protein
Chromosome I	1565355	1565382	+	GCCG**GTAA**AGGAATGGTTACCAAACGAG**CGAA**TTCTTA	BM28_RS07570	FliJ (ATPase complex)
Chromosome II	40104	40131	+	TTTG**GTAA**ATATGAAATGAATGCATGTA**CGAA**TGTTA	BM28_RS10500	*N*-Formylglutamate amidohydrolase
Chromosome II	69028	69051	–	CCAT**GTAA**GCGCGATGATGAATGG**CGAA**ACGGAC	BM28_RS10610	Lytic murein transglycosylase
Chromosome II	76323	76348	–	TTCGG**GTAA**GTTTCCATACATCACACG**CGAA**ACTTTC	BM28_RS10645	Hypothetical protein
Chromosome II	418369	418394	–	GCGAA**GTAA**AGCCAAGGCAATTTCTGA**CGAA**ATTGC	BM28_RS12275	Acetyl coenzyme A *C* acyltransferase
Chromosome II	726823	726850	+	CATC**GTAA**AGGAAAAGGAGCCGCGTAAG**CGAA**TGACCC	BM28_RS13730	DUF1127 domain-containing protein
Chromosome II	813509	813534	+	TTCC**GTAA**TTAATGAAAATCTATATA**CGAA**TCGGGGC	BM28_RS14145	Hypothetical protein
Chromosome II	1172859	1172884	+	TTTG**GTAA**AATTGCCAAACTTTTATG**CGAA**AACGG	BM28_RS15825	DhbR
Chromosome II	286089	286111	+	TACAATAACCCATTATAAGTAGA**CGAA**AAAAG	BM28_RS11665	NarR

a The boldface letters GTAA and CGAA indicate the conserved sequences of −35 and −10 motifs, respectively.

### Both BcrS and AbcS are required for full expression of T4SS.

**FIG 7:**
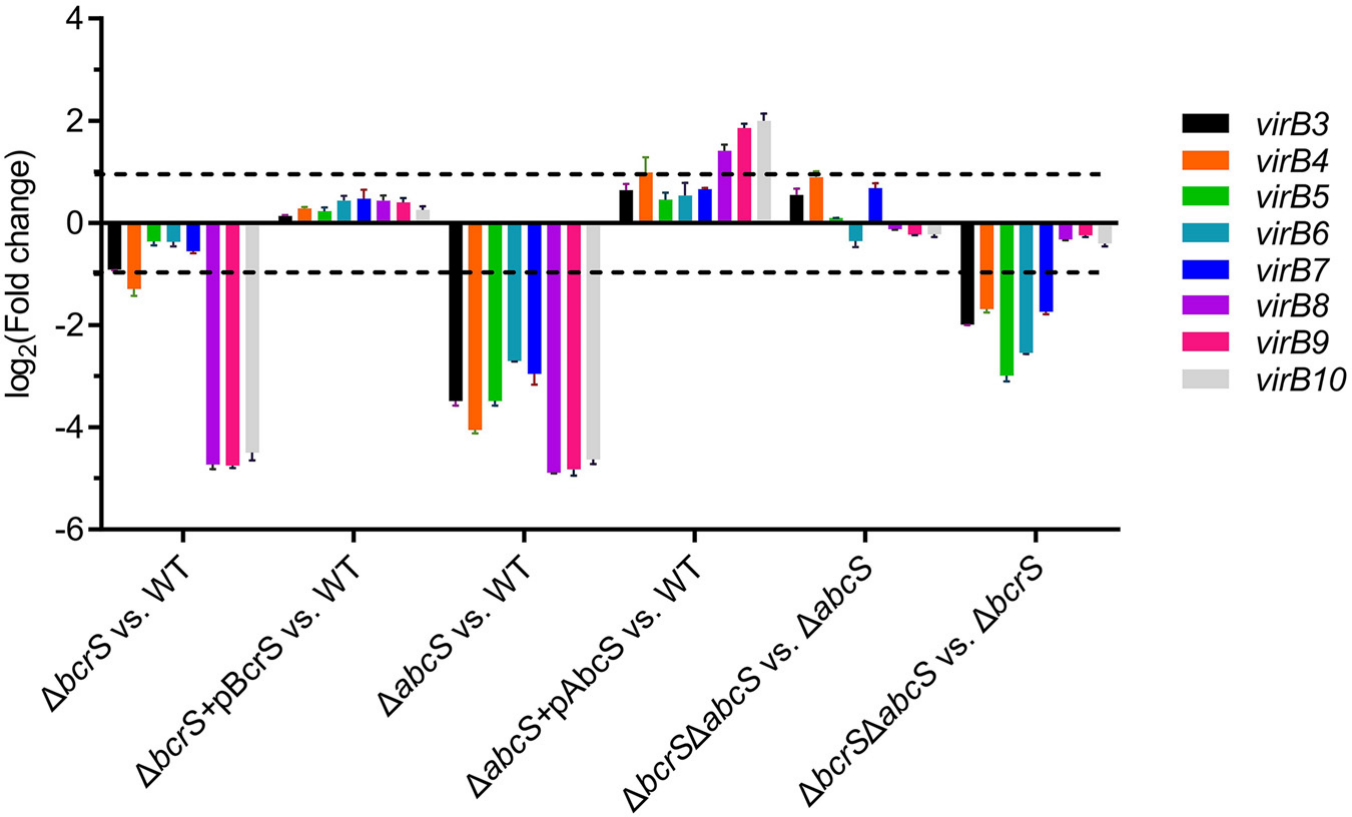
Contribution of *bcrS* and *abcS* to T4SS expression as assessed by qPCR. Bacteria were cultured in TSB broth until reaching an OD_600_ of ∼0.5 in the absence of HOCl, and then RNA samples were prepared. The 2^–ΔΔ^*^CT^* method was used to determine fold changes in each binary comparison indicated. The dashed lines denote a 2-fold change cutoff, and a fold change of ≥2 was considered significant. Values are the means ± the SEM from three independent experiments.

Commonly, ECF σ factors positively affect target gene expression; however, the role of an anti-σ in gene regulation can be multifaceted (43). To clearly delineate the impact of *bcrS* and *abcS* on T4SS expression, we used qPCR to evaluate the transcription levels of *virB3* to *virB10* genes in the wild type, in Δ*bcrS* and Δ*abcS* mutants, and in the Δ*bcrS* Δ*abcS* double mutant. As shown in Fig. 7, compared to the wild type, deletion of *bcrS* significantly reduced the expression of *virB3*, *virB4*, and *virB8* to *virB10*, while mutation of *abcS* impaired *virB3* to *virB10* expression. Moreover, transformation of a complementation plasmid with the deleted locus into the corresponding mutant markedly enhanced *virB3* to *virB10* expression. These results indicate that both BcrS and AbcS positively impact T4SS expression. Furthermore, in the Δ*abcS* mutant, a mutation of *bcrS* no longer reduced *virB3* to *virB10* expression; on the other hand, in the absence of *bcrS*, inactivation of *abcS* still caused a significant reduction in *virB3* to *virB7* expression. These data suggest that BcrS positively impacts T4SS expression in an AbcS-dependent manner, while AbcS can influence T4SS expression independently of BcrS.

### *B. melitensis* AbcS is required for the maintenance of chronic infection in a mouse model.

**FIG 8:**
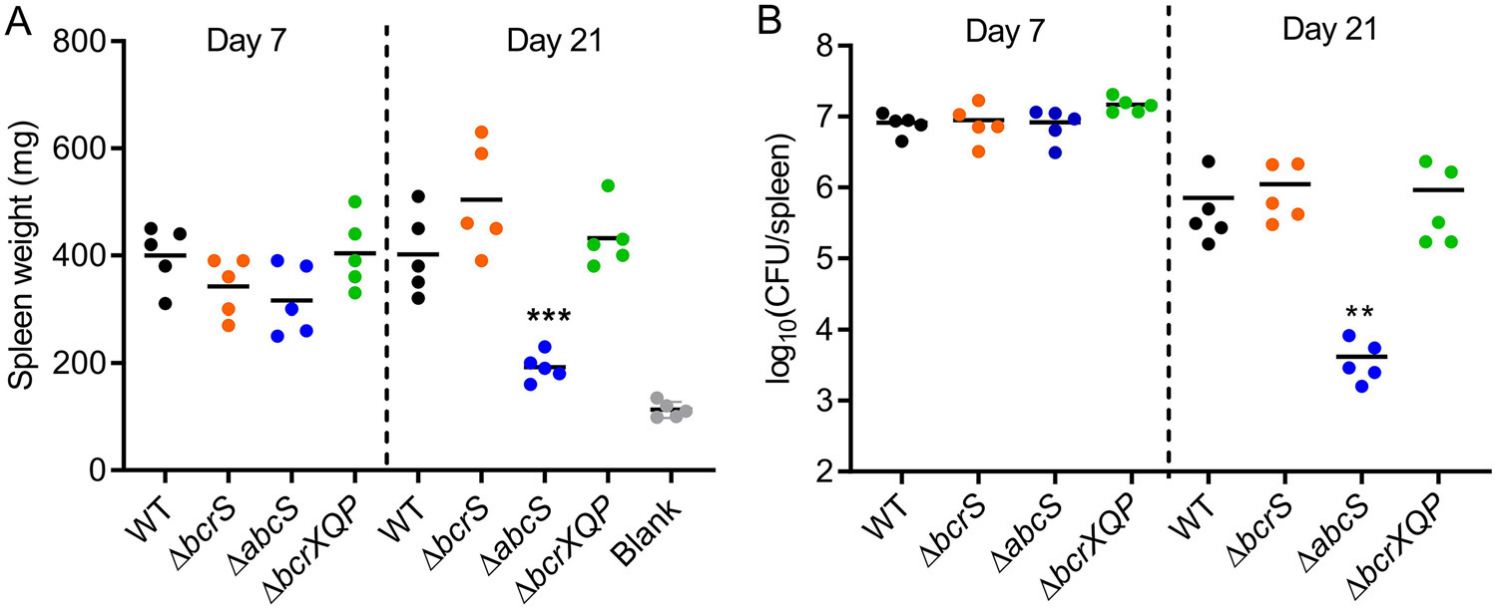
B. melitensis *abcS* is required for the maintenance of chronic infection in a mouse infection model. Six-week-old BALB/c mice were infected intraperitoneally with wild-type M28 and its various mutants. Spleen weights (A) and splenic bacterial burdens (B) were determined at 1 and 3 weeks postinfection. Bars amid data points in the graph denote the mean (for each time point, *n *=* *5 mice per bacterial strain). “Blank” indicates a control group of mice inoculated with PBS. **, *P* < 0.01; ***, *P* < 0.001 (one-way ANOVA, followed by Dunnett’s multiple-comparison test). This experiment was repeated twice, and representative data are shown.

To characterize the roles of *bcrS*, *abcS*, and *bcrXQP* in B. melitensis virulence *in vivo*, we tested the virulence of various mutants and the parental strain M28 in a mouse model. Groups of five BALB/c mice were intraperitoneally infected with M28 and its derivatives and then euthanized at 1 and 3 weeks after infection. Subsequently, the spleens were weighed out and bacterial splenic colonization was determined. As shown in Fig. 8, by 1 week postinfection, mice infected with the Δ*bcrS*, Δ*abcS*, and Δ*bcrXQP* mutants exhibited similar levels of splenomegaly and bacterial colonization relative to wild-type M28. Remarkably, compared to the wild type, the Δ*abcS* mutant displayed diminished bacterial load (*P* < 0.01) and splenomegaly (*P* < 0.001) at 3 weeks postinfection, whereas the spleen weights and bacterial colonization in mice infected with Δ*bcrS* and Δ*bcrXQP* mutants were comparable to those infected with the wild type. Thus, these data demonstrate that B. melitensis *abcS* is required for the maintenance of chronic infection but not for the establishment of an infection in mice.

## DISCUSSION

A domain-based BLASTP search of ECF σ in three major *Brucella* species, namely, B. melitensis, B. abortus, and B. suis, revealed that each genome contains two ECF σ factors: σ^E^ encoded by *rpoE1*, which is conserved in alphaproteobacteria (31), and a putative σ/anti-σ system, which is encoded by B0022/B0023 in B. melitensis M28. Orthologs of B0022 and B0023 in the type strain B. melitensis 16M were annotated as one single protein (BMEII0072, accession no. NC_003318), and an investigation of six σ factors in B. melitensis 16M showed that loss of BMEII0072 does not alter *Brucella* infection of murine macrophages but impairs chronic infection of mice (31), suggesting the importance of this genetic locus in *Brucella* virulence *in vivo*. In this study, we defined B0022/B0023 as an ECF16 cognate σ/anti-σ system and renamed them BcrS/AbcS. We characterized the regulons of BcrS/AbcS using RNA-seq and bioinformatic approaches and further described the regulatory details of BcrS/AbcS. Expression analysis demonstrated that AbcS contributes greatly to the expression of T4SS and serves as a critical virulence determinant, while BcrS is dispensable for virulence in the mouse model. Thus, this work has greatly improved our understanding of ECF16 σ/anti-σ in pathogenic bacteria.

Our data demonstrated that inactivation of *bcrS* reduced *bcrXQP* expression even in the absence of the inducer HOCl (Fig. 3D). Moreover, P*_bcrS_*-*lacZ* expression in the Δ*bcrS* mutant was much higher than the empty vector (Fig. 3C and 4B), suggesting that another σ factor may drive basal-level expression of *bcrS*. Similarly, the ECF16 prototype SigF in C. crescentus is expressed at basal levels and regulates target gene CC3255 expression, even in the absence of an exogenous inducer (37). Maintaining basal level expression of an ECF σ can be advantageous by ensuring bacteria a rapid response upon exposure to external stimuli.

We identified the recognition motifs of BcrS and established that BcrS directly activates *bcrXQP*. The Δ*abcS* mutant exhibited higher expression of *bcrS* and *bcrXQP* than the wild type (Fig. 4B and 3D). Hence, BcrS and AbcS regulate *bcrXQP* expression in a canonical fashion: BcrS is a positive regulator, while AbcS is an anti-BcrS factor. In contrast, both *bcrS* and *abcS* positively affect T4SS expression, and the positive role of *bcrS* relies on *abcS* because in the absence of *abcS*, *bcrS* no longer imposed positive effects on T4SS expression (Fig. 7). Thus, *abcS* has a “pro-sigma” role in regulating T4SS expression. Indeed, some anti-σ factors exhibit pro-σ activities. For instance, Escherichia coli FecR and Pseudomonas aeruginosa FoxR inhibit their cognate σ factors by anchoring them on the membrane in the absence of a signal, whereas the cognate σ factors FecI (44, 45) and FoxI (43) require FecR and FoxR to turn on the expression of the target genes when a signal is present. Mechanistically, it has been suggested that the N-terminal portion of an anti-σ interacts with its cognate σ and functions as a chaperone to promote σ protein stability (46). In addition, we found that *abcS* also affects T4SS expression in a *bcrS*-independent fashion (see Fig. S2 for a schematic model). Similarly, the anti-σ VreR of P. aeruginosa affects virulence gene expression independent of its cognate σ VreI (34). It is possible that by virtue of its structural capacity to bind σ_2_ and σ_4_, an anti-σ may regulate multiple σ factors, thus enabling cross talk (47) and playing a regulatory role independent of its cognate σ. Another interesting finding is that *virB* genes that are encoded in an operon were differentially regulated by BcrS, and this may be due to transcriptional attenuation, mRNA stability, and/or the involvement of posttranscriptional regulators. The molecular mechanisms underlying the regulation of T4SS expression by BcrS/AbcS warrant intensive future studies. To our knowledge, this is the first study to show that ECF16 anti-σ factors can have anti- and pro-sigma activities and regulate gene expression in σ-dependent and σ-independent manners.

In the bacterial two-hybrid assay, we found that only one combination of constructs (T18-AbcS and T25-BcrS) produced positive results. This may be attributable to (i) AbcS being a transmembrane protein with several hydrophobic segments, so fusing a protein to it should properly expose the interaction domain (otherwise an interaction will not occur), or (ii) E. coli being the bacterial host used in this assay, which failed to provide optimal cytosolic environments like that in the native host *Brucella*. BcrS and AbcS have relatively larger regulons (36 and 63 DEGs, respectively) than those of several other ECF16 σ members. Strikingly, genes encoding Msr are shared by at least three other ECF16 σ regulons (EcfF of Bradyrhizobium japonicum and SigF of *C. crescentus* (48), which both respond to ROS, and SigF of *A. suillum*, which responds to RCS), although these organisms lead distinct lifestyles. These commonalities imply that ECF16 σ-controlled Msr could be a very efficient mechanism in overcoming oxidative stresses. BcrS upregulates *bhuA*, which encodes a heme transporter, *dhbCE*, which is involved in 2,3-DHBA biosynthesis, and electron transporters (Fig. 5B and 6). These results suggest that BcrS plays a role in modulating *Brucella* fundamental physiology, such as iron utilization and electron transport. Indeed, previous reports have demonstrated the potential of ECFs in regulating siderophores (49) and energy metabolism (50).

We show that *bcrXQP* is considerably induced by HOCl and involved in HOCl resistance (Fig. 2 and 3C). One of the toxic effects imposed by HOCl is oxidation of proteins, especially conversion of a methionine to MetSO, which renders the proteins dysfunctional (51). Given that methionine residues in proteins can function as antioxidants (52), BcrX, similar to its homolog MrpX in *A*. *suillum* PS (39), could serve as an oxidative sacrificial sink protein that scavenges HOCl. MsrQ/MsrP in E. coli are homologous to M28 BcrQP, and the two proteins can repair HOCl-oxidized substrates, particularly the chaperone SurA and the lipoprotein Pal, via a mechanism in which MsrP catalyzes the reduction of MetSO to methionine through MsrQ, which derives electrons from the quinone pool of the respiratory chain (40). Since *bcrX*, *bcrQ*, and *bcrP* are cotranscribed and induced by HOCl (Fig. 3), we propose here that *bcrX* is the substrate of *bcrPQ* and that oxidized BcrX can be rescued through the putative Msr BcrP/BcrQ. In this manner, the *Brucella* cell envelope, including periplasmic proteins, can be protected from HOCl oxidation, and as a result, survival can be enhanced. HOCl is produced by both macrophages and neutrophils, and it is a primary mechanism used by neutrophils to kill microbes (53–56); therefore, *bcrXQP* may be specifically utilized by *Brucella* to increase survival within neutrophils. Thus, we initially predicted that a lack of *bcrS* or *bcrXQP* would lead to survival defects and consequently reduced colonization in mice. However, *bcrS* and *bcrXQP* are dispensable for M28 infection in the mouse model used here. In contrast, *abcS* plays a key role in M28 infection, specifically in the chronic phase of infection (Fig. 8). We speculate that this phenotype may be in large part associated with the great contribution of *abcS* to full expression of T4SS, which is required for chronic infection with *Brucella* in murine models (17, 57). RpoE1 in B. melitensis 16M and B. abortus 2308 also plays a critical role in chronic infection of mice (31, 32). Compared to B. melitensis BcrS in this study, B. abortus RpoE1 promotes bacterial survival during H_2_O_2_ and acid stress and regulates a different set of virulence genes, such as *rpoH1*, urease genes, and the lipopolysaccharide O-chain *ba14k* gene (32). Therefore, the *Brucella* BcrS/AbcS and RpoE1/NepR/PhyR systems seem to respond to different stress conditions and promote chronic infection using different mechanisms.

In summary, our work reveals the regulatory characteristics of an ECF16 σ/anti-σ system and suggests that *bcrS*-*abcS* links RCS resistance with T4SS expression. It is tempting to assume that with the help of the *bcrS*-*abcS* system, *Brucella* senses its presence within macrophages/neutrophils through the detection of HOCl and responds by activating *bcrXQP* expression for stress resistance, as well as T4SS expression, for evasion of host immune responses. Given that AbcS is crucial for virulence expression yet imposes no survival pressure, our study provides a robust therapeutic target to potentially combat chronic infections with *Brucella*.

## MATERIALS AND METHODS

### Bacterial strains and culturing conditions.

All strains and plasmids used in the study are listed in Table S6 in the supplemental material. B. melitensis M28 and its variants were grown on tryptic soy agar (TSA) or in TSB (Difco) at 37°C in a 5% CO_2_ atmosphere. All work with live *Brucella* was performed in a biosafety level 3 (BSL3) facility. E. coli strains (DH5α and strains used in the bacterial two-hybrid system) were grown in Luria-Bertani (LB) medium at 37°C. When needed, antibiotics were added at the following concentrations: kanamycin, 50 μg/ml; ampicillin, 100 μg/ml; and chloramphenicol, 25 μg/ml (10). Hypochlorous acid (HOCl) was added to the medium at the indicated concentrations, and a concentration of up to 2 mM does not alter the pH in medium (data not shown).

### Construction of *Brucella* mutants and recombinant plasmids.

In-frame deletion mutants of *bcrS*, *abcS*, and *bcrXQP* in B. melitensis M28 were constructed by homologous recombination as previously described (58, 59). Briefly, upstream and downstream regions ∼1,000 bp in length of the gene of interest were PCR amplified from the B. melitensis M28 genome, which was followed by cloning the two fragments to flank a kanamycin resistance cassette in the pSP72 suicide vector (Amp^r^) using a MultiS one-step cloning kit (Vazyme, China). The resulting recombinant plasmid was transformed into M28 electrocompetent cells, and the transformants were selected on TSA supplemented with kanamycin. Candidate deletion mutants, which were resistant to kanamycin but sensitive to ampicillin, were further subjected to PCR amplification and Sanger sequencing for validation. The *bcrS* complemented strain was generated by transforming into the Δ*bcrS* mutant a pBBR1MCS4 variant carrying the *bcrS* locus driven by its native promoter (in qPCR analysis of *bcrXPQ* expression) or P_KmR_ promoter (in BcrS autoregulation study), while the *abcS* complemented strain was generated by transforming into the Δ*abcS* mutant a pBBR1MCS4 variant carrying the a*bcS* locus driven by the P_KmR_ (from pHD-kan, accession number KF947529.1) constitutively expressed promoter.

The pBBR-*lacZ* vector plasmid and its derivatives used for transcriptional fusion were constructed according to a previously reported protocol (60, 61). Briefly, the *lacZ* coding region, along with its 5′-leader sequence from pVIK112 (62), was cloned into pBBR1MCS4 using SphI and SacI, resulting in pBBR-*lacZ*. Regions of ∼500 bp containing the promoters of *bcrS* and *bcrXQP* were PCR amplified from the M28 genome and cloned into pBBR-*lacZ* using EcoRI and XbaI sites, respectively, thereby producing the promoter-*lacZ* fusion plasmids pBBR-P*_bcrS_*-*lacZ* and pBBR-P*_bcrXQP_*-*lacZ*. These plasmids were transformed into wild-type M28 and its mutants by electroporation to monitor the expression of P*_bcrS_*-*lacZ* and P*_mrpXQP_*-*lacZ*, respectively. Similarly, pBBR-P_KmR_-*lacZ* was constructed and used as a constitutively expressed *lacZ* control. All plasmid constructs were confirmed by DNA sequencing to ensure correctness. The empty vector did not affect the experiments we performed in this study (63–65). All of the primers used in this study are listed in Table S7.

### Bacterial two-hybrid system.

To explore the interactions between BcrS and AbcS, the BATCH system based on the Bordetella pertussis adenylate cyclase T25 and T18 fragments was used according to the vendor’s instructions (EUK001; Euromedex). Translational fusions to T25 at its N and C termini were constructed by ligating CDS regions into pKNT25 and pKT25 plasmids, respectively, using XbaI and EcoRI sites, and translational fusions to T18 at its N and C termini were constructed by ligating CDS regions into pUT18 and pUT18C plasmids, respectively, using XbaI and EcoRI sites. All plasmid constructs were verified by DNA sequencing. Different combinations of plasmids, as illustrated in Fig. 1C, were cotransformed into the BTH101 host strain, and the resultant transformants were spotted onto LB agar plates containing 100 μg/ml X-Gal (5-bromo-4-chloro-3-indolyl-β-d-galactopyranoside), 0.5 mM IPTG (isopropyl-β-d-thiogalactopyranoside) and appropriate antibiotics to assay the β-galactosidase activities. A combination of plasmids pKT-Zip and pUT-Zip was used as a positive control, and empty vectors were used as negative controls. Blue colonies indicate substantial β-galactosidase activities, suggesting significant protein interactions, whereas white colonies indicate very low β-galactosidase activities, suggesting no detectable protein interactions (66).

### RNA isolation, RT-PCR, and qPCR.

RNA extraction and reverse transcription-PCR (RT-PCR) and quantitative reverse transcription-PCR (qPCR) analyses were performed as previously described (58, 59). B. melitensis M28 and the derivative mutants were cultured in TSB medium at 37°C until reaching an optical density at 600 nm (OD_600_) of ∼0.5, and then equal portions of a bacterial culture were collected and treated with the inducers HOCl (1 mM) and H_2_O_2_ (1 mM) or phosphate-buffered saline (PBS) as a control. After incubation for 10 min with shaking, the cultures were immediately mixed with RNAprotect reagent (Qiagen) and subjected to RNA extraction by TRIzol reagent according to the manufacturer’s instructions (Invitrogen). RNA samples were then treated to remove genomic DNA and reverse transcribed to cDNA using a PrimeScript RT reagent kit with gDNA Eraser (Clontech).

For the cotranscription test by RT-PCR, primer pairs were designed to span *bcrP* and *bcrQ*, *bcrQ* and *bcrX*, *bcrP* to *bcrX*, and *bcrS* and *abcS*, yielding amplicons of ∼850, ∼700, ∼1,900, and ∼620 bp, respectively. RNA was extracted as described above from M28 grown in TSB. RNA that was not reverse transcribed served as a negative control, while genomic DNA served as a positive control (67).

For qPCR, cDNA was used as a template for SYBR green-based qPCRs using TB Green Premix *Ex Taq* II Reagent (Clontech) and an ABI Quant 5 thermocycler (Applied Biosystems). Melting curve analyses were performed after each reaction to ensure amplification specificity. Fold changes in transcript levels were calculated using the 2^−ΔΔ^*^CT^* method (68), and levels were normalized according to 16S rRNA expression. Differences between groups were analyzed by Student *t* test.

### 5′-RACE PCR analysis to identify transcription start sites.

The TSSs of *bcrS* and *bcrXPQ* were identified using the SMARTer 5′/3′-RACE kit (Clontech) according to the manufacturer’s manual (69). RNA from wild-type M28 was isolated as described above. Complete removal of DNA contamination was confirmed using RT-PCR. Approximately 5 μg of RNA was reverse transcribed with gene-specific primers, and nested PCR was performed to obtain the PCR products, which were subsequently cloned into the pRACE vector provided with the kit. Multiple constructs were selected and subjected to DNA sequencing and sequence analysis to identify the TSS.

### RNA-seq.

RNA-seq analysis was performed using a standard protocol with minor modifications (70). Briefly, RNA extractions were carried out as described above, and then the quality and concentrations were determined by an Agilent 2100 Bioanalyzer (Agilent Technologies) and NanoDrop system (Thermo Fisher Scientific, Inc.), respectively. One microgram of high-quality RNA (*A*_260_/*A*_280_ ratio > 2.0 and RIN value > 7.0) was used for each NextGen sequencing library, which was constructed according to the manufacturer’s protocol (NEBNext Ultra Directional RNA Library Prep kit for Illumina). rRNA was depleted from total RNA using the Ribo-Zero rRNA removal kit for bacteria (Illumina, CA). Sequencing of the libraries was performed using a 2 × 150 paired-end configuration on an Illumina HiSeq platform according to the manufacturer’s instructions (Illumina), and image analysis and base calling were conducted using HiSeq Control Software (HCS) + OLB + GAPipeline-1.6 (Illumina) on a HiSeq system. The sequences were processed and analyzed, and raw reads were assessed by fastQC and further treated by Cutadapt (version 1.9.1). Clean reads were then aligned to the B. melitensis M28 genome (GenBank accession numbers NC_017244.1 and NC_017245.1) using bowtie2 (version v2.1.9, standard options). Reads were counted using Htseq (v0.6.1). Differential gene expression analysis was then performed using DESeq2 (v1.6.3) with R version 3.3.2 following a standard workflow. All genes with a |log_2_(fold change)| > 1 and a Benjamini-Hochberg adjusted *P* value (*q* value) of <0.05 were considered differentially expressed. Tables S1 to S5 list differentially expressed genes in the five groups, and raw data are available at the National Microbiology Data Center (http://nmdc.cn/; accession no. NMDC40002004 to NMDC40002009).

### HOCl survival assays.

HOCl survival assays were performed as previously described (40). All *Brucella* strains were grown on TSA for 72 h, and cells were then harvested and resuspended in sterile PBS at an OD_600_ of ∼0.02. Bacterial suspensions were diluted 1:100 into 10 ml of TSB and allowed to grow until reaching an OD_600_ of ca. 0.4 to 0.5. The OD_600_ values were normalized, and two equal portions were taken, with one portion treated with HOCl at a final concentration of 2 mM and the other with PBS as a control. These cultures were then incubated at 37°C with shaking at 210 rpm, collected at various time points, and spotted onto TSA to determine the CFU counts. The CFU counts at time point 0 were set as 100%. Survival rates were determined as (CFU at a time point after stress/CFU at time point 0) × 100%.

### β-Galactosidase activity assay.

All bacterial strains carrying *lacZ* transcriptional fusions were grown overnight in TSB at 37°C, and then the cultures were diluted 1:100 into 10 ml of fresh TSB and allowed to grow until reaching an OD_600_ of ca. 0.4 to 0.5. Multiple equal portions of bacterial cultures were collected and treated with HOCl at the indicated concentrations or with PBS as a control. These cultures were then incubated at 37°C with shaking for 60 min before being subjected to β-galactosidase activity measurements as previously reported (61, 71).

### Mouse infection assay.

Assessment of B. melitensis virulence in BALB/c mice was conducted in a BSL3 facility as previously described (58, 59). Briefly, groups of five 6-week-old mice with similar weights were challenged via an intraperitoneal route with 100 μl of bacterial culture containing 1 × 10^6^ CFU of M28 or its mutant strains. At 1 and 3 weeks postinfection, mice were sacrificed by cervical dislocation, and spleens were aseptically removed, weighed, and homogenized in PBS containing 0.1% Triton X-100. Spleen homogenates were serially diluted 10× in PBS and plated on TSA with appropriate antibiotics to determine the bacterial loads, which are expressed as log_10_CFU per spleen.

### Statistical analysis.

One-way analysis of variance (ANOVA), followed by Dunnett’s multiple-comparison test, was used to analyze the differences between various mutants and the wild-type strain (GraphPad 9.0; Prism), and all other binary comparisons were analyzed by a Student *t* test. A *P* value of <0.05 was considered statistically significant.

### Ethics statement.

Handling and care of mice were performed according to the Beijing Administration Guidelines for the Use of Laboratory Animals. The entire protocol with respect to animal experiments was approved by the Review Board of Harbin Veterinary Research Institute and by the Animal Care and Use Committee of Heilongjiang Province [SYXK(H)2006-032]. Procedures regarding the infection of BALB/c mice with *Brucella* were approved by the Chinese Ministry of Agriculture.

### Data availability.

The data that support the findings of this study are openly available at the National Microbiology Data Center (http://nmdc.cn/; accession no. NMDC40002004 to NMDC40002009).
