# Supplementary material for: An Extracytoplasmic Function Sigma/Anti-Sigma Factor System Regulates Hypochlorous Acid Resistance and Impacts Expression of the Type IV Secretion System in Brucella melitensis
Source: J Bacteriol. 2021 May 20;203(12):e00127-21. doi: 10.1128/JB.00127-21 (PMC8315932; doi:10.1128/JB.00127-21)
Supplement: Supplementary file 4 [file jb.00127-21-s0004.pdf]

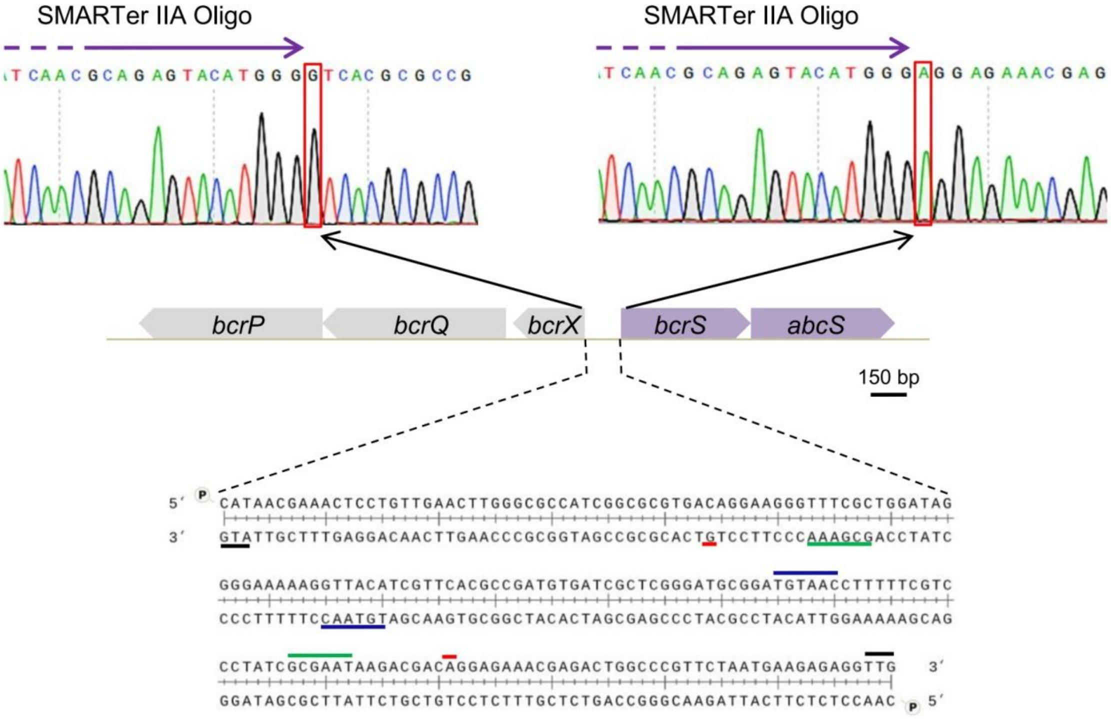

FIG S1 Identification of transcription start sites (TSS) of *bcrXQP* and *bcrS*/*abcS*. The chromatograms indicate the DNA sequencing results, with the arrows showing the adaptor SMARTer IIA Oligo and boxed letters as the TSSs. The -10 and -35 motifs were deduced afterwards. Black, red, green, and blue lines amid the DNA sequence represent start codon, TSS, -10 and -35 motif, respectively. Genes were drawn to scale.

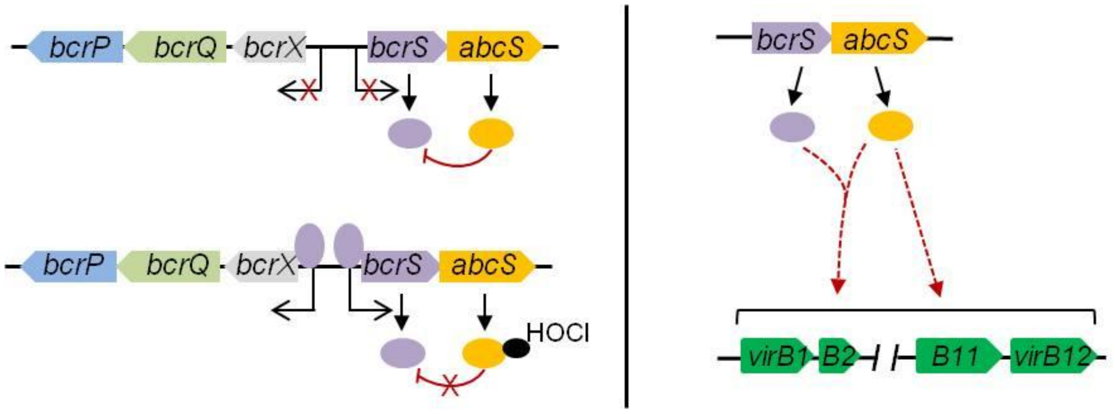

FIG S2 A schematic model depicting the regulation of *bcrXPQ* (left) and *virB* genes (right) by BcrS and AbcS. Red arrows indicate positive (pointed arrows) or negative regulation (blunt arrows); dashed arrows indicate regulation with unknown mechanisms; red crosses indicate a blocking effect; bend arrows indicate promoter direction and activities.
